# Particular matter influences the incidence of acute otitis media in children

**DOI:** 10.1038/s41598-021-99247-3

**Published:** 2021-10-05

**Authors:** Mina Park, Jiyeon Han, Jiwon Park, Myoung-jin Jang, Moo Kyun Park

**Affiliations:** 1 Department of Otorhinolaryngology-Head and Neck Surgery, Seoul Medical Center, Seoul, South Korea; 2 Medical Research Collaborating Center, Seoul National University Hospital, Seoul, South Korea; 3 Department of Otorhinolaryngology-Head and Neck Surgery, Sensory Organ Research Institute, Seoul National University Medical Research Center, Seoul National University College of Medicine, 101 Daehangno, Jongno-gu, Seoul, South Korea

**Keywords:** Environmental sciences, Environmental social sciences, Diseases, Medical research, Risk factors

## Abstract

Particulate matter (PM) is the main component of air pollution. Children are vulnerable to PM and acute otitis media (AOM), which is one of the most common diseases in children. However, studies on the relationship between AOM in children and PM are rare and their results are inconsistent. The aim of this study is to investigate the effect of PM on AOM in children on the basis of the Korea National Health Insurance service (NHIS) claims data. NHIS claim data from 2008 to 2015 was used to identify outpatient visits, antibiotic use to treat AOM, and demographic data. This data was combined with the data on PM_2.5_ (≤ 2.5 μm) and PM_10_ (≤ 10 μm according to its aerodynamic diameter) level extracted from air pollution data from Korean National Institute of Environmental Research for 16 administrative regions. The children with AOM were divided into three age groups (< 2, 2–4, 5–10 years). Generalized linear Poisson regression model was used to estimate the association between AOM and PM using daily counts of AOM and daily mean PM concentrations. It was adjusted to temperature, wind, humidity, season, year, age, and region. With an increase in PM_2.5_ of 10 μg/m^3^, the relative risk of OM increased by 4.5% in children under 2 years of age. The effect of PM_2.5_ was strongest influence on the day of exposure. The exposure to PM_10_ was related to the incidence of AOM on the day of exposure and the following seven days in all three age groups. The PM concentrations did not strongly affect either AOM duration or the use of antibiotics to cure AOM. The RR in the each lag day after exposure to PM_10_ was diverse according to the age groups. Regardless of PM size and children’s age, the PM levels are positively related to the incidence of AOM. Both PM_2.5_ and PM_10_ have the most adverse effects on children under 2 years of age and on the day of exposure.

## Introduction

Particulate matter (PM) is an important component and key indicator of air pollution. There has been growing concern about the effect of exposure to PM on public health. PM comes mostly from vehicles, industry, combustion, and natural sources. The World Health Organization defines PM as a class I carcinogen, and a global exposure mortality model has estimated that 8.9 million deaths are related to PM exposure in 2015^1^. PM enters and is absorbed by the human body through the respiratory system and affects the whole body systemically. It is a risk factor for the development of pulmonary, cardiovascular, cutaneous, metabolic, and neurocognitive diseases^2–5^. It is harmful for human health, especially in the elderly, pregnant women, and children^5,6^. Moreover, it increases the frequency of hospital admissions and emergency room visits. PM can be classified according to particle size into coarse (PM_10_, aerodynamic diameter ≤ 10 µm) and fine (PM_2.5_, aerodynamic diameter ≤ 2.5 µm). PM_2.5_ is more harmful for health due to its small size and easy systemic penetration^7^.

AOM is an important health problem in terms of prevalence and medical cost. On the basis of the National Health Insurance System (NHIS) data, Kim et al. reported an AOM prevalence of 152.7 in 2012 and 137.4 in 2017 in children aged 0–12 years in Korea. This means that 1.3–1.5 among 10 children suffer from AOM^8^. The costs related to OM are estimated at USD 3.2 billion annually and it is one of the five most costly conditions in children in the USA^9^.

AOM cause conductive hearing loss in children and adults^10^. This delays the development of speech, language, balance, and learning abilities^11^. OM significantly impacts the quality of life of children and their families. In addition, its adverse effects include sleep disturbance, loss of appetite, and behavioral problems^12^. Therefore, identification and control of risk factors for OM are important for global healthcare in terms of quality of life and medical costs. There is growing evidence for the association between PM and OM development^13–15^.

AOM is one of the most common diseases in young children^16,17^, who are very vulnerable to PM exposure^5,18^. For this reason, we investigated the association between AOM and PM in children. The number of AOM cases is sufficient to determine the daily incidence and regional differences. Diagnosis is well defined and relatively clear via otoscope examination compared to upper respiratory diseases^19^. Claim data of the NHIS, which all Koreans are affiliated with, includes age, gender, diagnosis, and daily medications^20^. Our previous study, which used a national sample cohort and investigated 0.16 million clinic visits, has shown that, among air pollutants, PM has the greatest influence on OM development^13^. However, that study had weekly temporal resolution because there were not enough cases for daily analysis, and spatial resolution for different urban and rural areas was low. Furthermore, it did not include children younger than 5 years of age.

The treatment choices for AOM comprise the use of antibiotics and ‘careful waiting’, which was first introduced by the American Academy of Pediatrics (AAP) and American Academy of Family Physicians (AAFP) in 2004^21^. In South Korea, the ‘Korean clinical practice guidelines: otitis media in children’ were developed in 2010 and revised in 2014 by the Korean Otology Society^22^. In both the America and South Korea, the guidelines have been modified to increase the proportion of careful waiting and to limit the use of antibiotics only to severe cases^17^. As far as we know, there are no large-scale national studies to reveal the association between PM and the use of antibiotics.

In this study, we used NHIS claim data for the whole country to include younger children and to investigate the daily incidence and improve spatial resolution of different areas. We also evaluated the disease duration and antibiotic use to treat AOM according to the concentration of PM.

## Materials and methods

### Database

To satisfy the medical demand, South Korea government established the NHIS, which is available to all people since 1989. It has general data including diagnostic codes and prescriptions, and has opened these data (from 2002) to the public in 2007^23^. We used the AOM and PM data from 2008 to 2015.

### Study sample and study design

**Figure 1:**
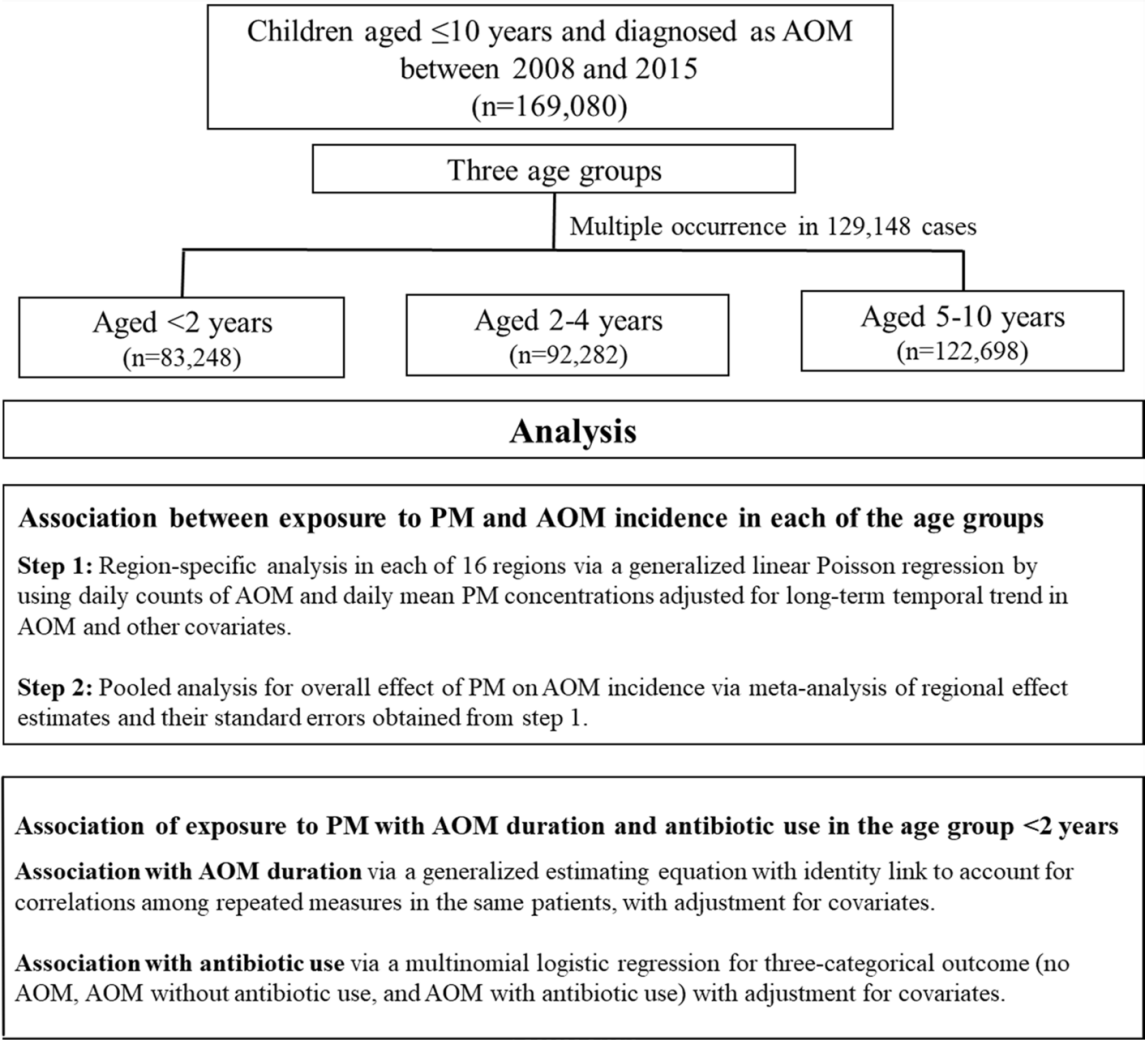
Flow diagram of this retrospective study.

The National Sample Cohort (NSC) is composed of 1 million nationally representative people, about 2% of the whole people of 50 million. This research used this NSC like our previous study^13^. The study flow diagram is shown in Fig. 1.

### AOM

The AOM patients who visited clinics or hospitals were identified on the basis of the International Classification of Disease, 10th edition (ICD-10) codes H65.0 (acute serous OM), H65.1 (other acute nonsuppurative OM), and H66.0 (acute suppurative OM)^24^. A total of 169,080 children were included in this study and they were subdivided into three age groups: < 2 (n = 83,248), 2–4 (n = 92,282), and 5–10 years (n = 122,698). For the analysis of the effect of PM levels on the incidence of AOM, we used the numbers of individuals, not the counts of clinic visits, because one child could visit the clinic several times. The analysis of the effect of PM levels on AOM disease duration was conducted for the < 2 year-old group only. To evaluate the effect of urbanization on AOM, metropolitan cities in terms of administrative region (Gwangju, Daegu, Daejeon, Busan, Seoul, Ulsan, and Incheon) were classified as urban and the others areas as rural. Disease duration assessment was based on the given and end day of disease code. Considering multiple occurrences in the same individual, it was documented with in the number of cases (n = 77,164). Antibiotic use to treat AOM was estimated from the prescription of main components of antibiotics, in addition to the disease code. A total of 57 different antibiotics were used that belonged to four categories: beta-lactam antibiotics (20), cephalosporins (9), macrolides (22), and special antibiotics (6) for resistant organisms according to the culture results.

This study was approved by the Institutional Review Board of the Seoul National University Hospital (1509-056-702).

### Particulate matter

Korean National Institute of Environmental Research releases regional air pollution data to the public on its official website, AirKorea^25^. It includes several air pollutants, however, in this study, we used PM ≤ 2.5 μm and ≤ 10 μm according to its aerodynamic diameter (PM_2.5_ and PM_10_, respectively, as determined by a β-ray absorption method). The number of total monitoring stations was 313, which were installed in 79 areas in 16 administrative regions. The concentrations of PM were measured hourly and the daily averages were used in this study. We used the data between 2008 and 2015 study. We divided the data into subgroups according to the PM_2.5_ concentrations (< 16, 16–36, 36 ≤ μm/m^3^) for the analysis of disease duration and antibiotics use. We used weather data for temperature, humidity, and wind speed per hour obtained from the Korea Meteorological Administration (https://www.kma.go.kr/kma/archive/pub.jsp)^26^; the daily levels were calculated by averaging hourly data.

### Statistical analysis

The association between AOM and exposure to PM was examined in each of the three age groups (< 2, 2–4, and 5–10 years). For each region, we used a generalized linear Poisson regression model to estimate the association between AOM and PM by using daily counts of AOM and daily mean PM concentrations. We adjusted for the long-term temporal trend in AOM with a natural cubic regression spline of 7 degrees of freedom (df) per year, 4-day average temperature with a natural cubic spline, and humidity as a continuous variable. A quasi-Poisson distribution was assumed to account for over-dispersion in the daily AOM counts. Delayed effects of PM on AOM were estimated for lags of 0–7 days. The net effect of PM for up to 7 days and the sum of the effects for all 8 days were presented along with delayed effect estimates. Relative risk (RR) results were presented in 10 μg/m^3^ increments for PM. The overall effects of PM were estimated using meta-analysis of regional effect estimates and their standard errors. Pooled results were shown both with random-effects models via the DerSimonian and Laird method and fixed-effects models via the inverse variance method. Statistical heterogeneity across studies was assessed with the χ^2^ test and the I^2^ statistics. Results from fixed-effects models were presented as primary pooled results if substantial heterogeneity was not found (I^2^ < 50%); otherwise, a random-effect model was applied.

The association between AOM and exposure to PM according to sex in each age group could not be analyzed in each region because the number of daily AOM cases was too small for such analysis. We were able to perform this analysis only for the two largest regions (Gyeonggi and Seoul).

The effects of PM concentration on AOM duration and antibiotic use were examined in the age group of < 2 years. For this analysis, individual data were used instead of aggregated data and the following confounders or covariates were adjusted for: sex, age, region, season, year, daily precipitation, daily temperature difference, daily minimum relative humidity, and daily wind speed (daily maximum wind speed for AOM duration and daily average wind speed for antibiotic use). Each of the daily temperature, relative humidity, and wind speed covariates was the one that had most significant relationship in univariate analysis among minimum, maximum, average and difference per day for temperature, between minimum and average for relative humidity, and between maximum and average for wind speed. Sensitivity analyses were performed by changing the degrees of freedom (df) of long-term trends (df = 6, 7, and 8) and by including a seasonality term (spring, summer, fall, and winter) as a categorical covariate in the Poisson regression model. For the association between the duration of AOM and PM, a generalized estimating equation with identity link was used to account for correlations among repeated measures in the same patient. For association with antibiotic use, a multinomial logistic regression was performed for the categorical outcome (no AOM, AOM without antibiotic use, and AOM with antibiotic use). Odds ratios (ORs) for AOM without and with antibiotic use relative to no AOM were presented for the association between PM and AOM antibiotic use. Statistical analyses were performed using SAS Enterprise Guide (ver. 7.13; SAS Institute, Cary, NC, USA) and R version 3.3.3.

## Results

### Incidence of AOM and levels of PM according to the regions

During the 8 years of this study (2008–2015), the daily national AOM incidence was 2.9 per 1,000 children, and the incidence was higher in children under 4 years of age than in those over 5 years of age. The daily concentration of PM_2.5_ and PM_10_ varied depending on the regions and was highest in Gyeonggi (55.4 ± 30.6) and Jeonbuk (34.3 ± 20.2), and lowest in Jeonnam (40.3 ± 22.8) and Gyeongbuk (21.8 ± 12.1) (S. Table 1).

### Association between PM and of AOM according to age in children

**Figure 2:**
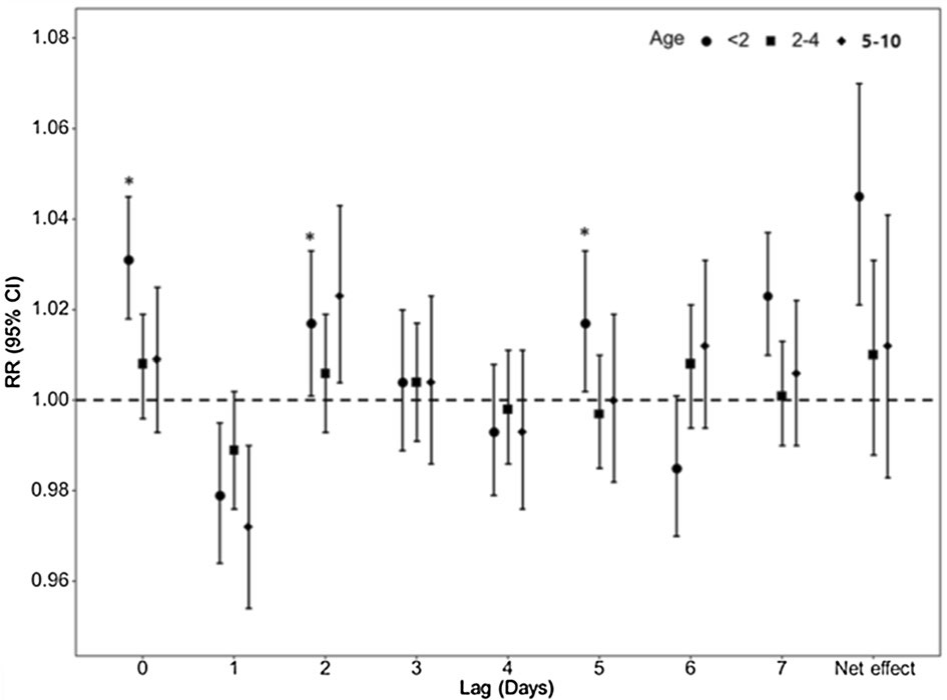
Relative risks of PM_2.5_ for acute otitis media according to lag times in three age groups. Lag 0–7 means the days prior to acute otitis media.

As can be seen from the *net effect* in Fig. 2, among children under 2 years of age, an increase in PM_2.5_ was associated with a significant increase in the number of AOM cases on the day of exposure and the subsequent 7 days. The pooled result of 16 region-specific effect estimates was a 1.045 increase in AOM case numbers over the 8 days for each 10 μg/m^3^ increase in PM_2.5_ (RR = 1.045, 95% CI = 1.021–1.070) (Table 2), and region-specific effect estimates ranged from 0.952 to 1.229 with a heterogeneity of 25% (S. Fig. 1). Statistically significant increases in AOM case numbers were found on the exposure day (lag 0) and the second (lag 2) and fifth (lag 5) days after exposure (Fig. 2). There was no significant difference in the effect of PM_2.5_ on AOM on the day of exposure (lag 0) between urban and rural areas (RR = 1.041, 95% CI = 1.019–1.063; RR = 1.025, 95% CI = 1.007–1.043; S. Fig. 2). The association of PM_2.5_ with AOM was not significant among 2–4 year-old children (RR = 1.010, 95% CI = 0.988–1.031) and those of ≥ 5 years of age (RR = 1.012, 95% CI = 0.983–1.041) (Table 1). When the data from Gyeonggi and Seoul were used for analysis according to sex and age groups, the pooled results for PM_2.5_ were 1.039 (95% CI = 0.992–1.087) for boys and 1.062 (95% CI = 0.943–1.196) for girls (Table 2). Sensitivity analyses showed that the effect of PM on AOM remained significant for different long-term trends and seasonality (Table 3).

**Table 1:** The relationship between the PM and the relative risks of acute otitis media according to the three age group.

	PM_2.5_			PM_10_		
	RR*	95% CI		RR*	95% CI	
< 2 yrs	1.045	1.021	1.070	1.007	1.003	1.011
2–4 yrs	1.010	0.988	1.031	1.009	1.006	1.013
5–10 yrs	1.012	0.983	1.041	1.006	1.002	1.010

*PM*_*2.5*_ particulate matter ≤ 2.5 μm/m^3^, *RR* relative risk, *CI* confidence interval, *yrs* years.

*Net effect of PM up to 7 days after exposure.

**Table 2:** Results by sex and age groups using data from two regions Gyeonggi and Seoul.

	PM_2.5_			PM_10_		
	RR*	95% CI		RR*	95% CI	
**< 2 yrs**						
Boys						
Gyeonggi (rural)	1.042	0.985	1.101	1.008	0.998	1.017
Seoul (urban)	1.032	0.950	1.120	1.009	0.996	1.023
Pooled	1.039	0.992	1.087	1.008	1.000	1.016
Girls						
Gyeonggi (rural)	1.002	0.941	1.067	1.007	0.997	1.018
Seoul (urban)	1.131	1.044	1.226	0.996	0.983	1.009
Pooled	1.062	0.943	1.196	1.003	0.995	1.011
**2–4 yrs**						
Boys						
Gyeonggi (rural)	1.004	0.946	1.066	1.012	1.004	1.020
Seoul (urban)	1.040	0.961	1.127	1.009	1.000	1.019
Pooled	1.017	0.970	1.067	1.011	1.005	1.017
Girls						
Gyeonggi (rural)	1.051	0.989	1.117	1.014	1.006	1.023
Seoul (urban)	1.039	0.967	1.117	1.012	1.002	1.021
Pooled	1.046	0.998	1.096	1.013	1.007	1.019
**5–10 yrs**						
Boys						
Gyeonggi (rural)	1.042	0.974	1.115	1.014	1.005	1.024
Seoul (urban)	0.942	0.852	1.041	1.004	0.992	1.016
Pooled	0.998	0.905	1.100	1.010	1.003	1.018
Girls						
Gyeonggi (rural)	1.008	0.936	1.086	1.010	1.001	1.020
Seoul (urban)	1.123	1.031	1.223	1.003	0.992	1.015
Pooled	1.062	0.956	1.180	1.007	1.000	1.015

*PM*_*2.5*_ particulate matter ≤ 2.5 μm/m^3^, *RR* relative risk, *CI* confidence interval, *yrs* years.

*Net effect of PM up to 7 days after exposure.

**Table 3:** Results of sensitivity analysis for different long-term trends and seasonality.

	PM_2.5_			PM_10_		
	RR	95% CI		RR	95% CI	
**< 2 yrs**						
M with df = 7	1.045	1.021	1.070	1.007	1.003	1.011
S1 (M with df = 7 + seasonality)	1.042	1.018	1.067	1.007	1.003	1.011
S2 (M with df = 6)	1.068	1.030	1.108	1.010	1.006	1.014
S3 (M with df = 8)	1.048	1.024	1.073	1.006	1.002	1.010
**2–4 yrs**						
M with df = 7	1.010	0.988	1.031	1.009	1.006	1.013
S1 (M with df = 7 + seasonality)	1.001	0.979	1.023	1.010	1.006	1.013
S2 (M with df = 6)	1.012	0.991	1.033	1.011	1.008	1.015
S3 (M with df = 8)	1.017	0.996	1.039	1.008	1.005	1.012
**5–10 yrs**						
M with df = 7	1.012	0.983	1.041	1.006	1.002	1.010
S1 (M with df = 7 + seasonality)	1.010	0.981	1.040	1.007	1.003	1.011
S2 (M with df = 6)	1.016	0.988	1.045	1.007	1.003	1.011
S3 (M with df = 8)	1.012	0.983	1.041	1.006	1.002	1.010

**Figure 3:**
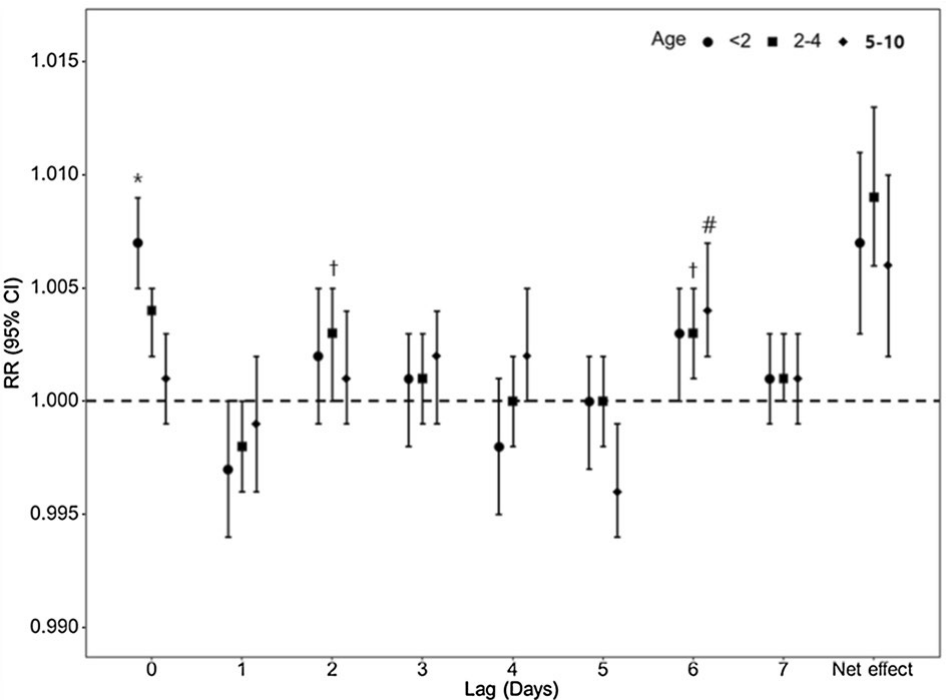
Relative risks of PM_10_ for acute otitis media according to lag times in three age groups. Lag 0–7 means days prior to acute otitis media. Three marks (*, †, #) indicate significant increases.

As can be seen from the *net effect* is shown in Fig. 3, the increase in PM_10_ was associated with the increase in AOM on the day of exposure and the subsequent 7 days in all three age groups. The pooled results for each 10 μg/m^3^ increase in PM_10_ were a 1.007 increase in AOM in 8 days among children aged < 2 years (RR = 1.007, 95% CI = 1.003–1.011, I^2^ = 29), a 1.009 increase among those aged 2–4 years (RR = 1.009, 95% CI = 1.006–1.013, I^2^ = 23), and a 1.006 increase among those aged ≥ 5 years (RR = 1.006, 95% CI = 1.002–1.010, I^2^ = 24) (Table 1). Significant increases in AOM case numbers were found on the day of exposure among children aged < 2 years, with the increments on the second and sixth days after exposure among those aged 2–4 and on the sixth day among those aged 5–10 years (Fig. 3).

### AOM duration and antibiotic use according to the levels of PM_2.5_

**Figure 4:**
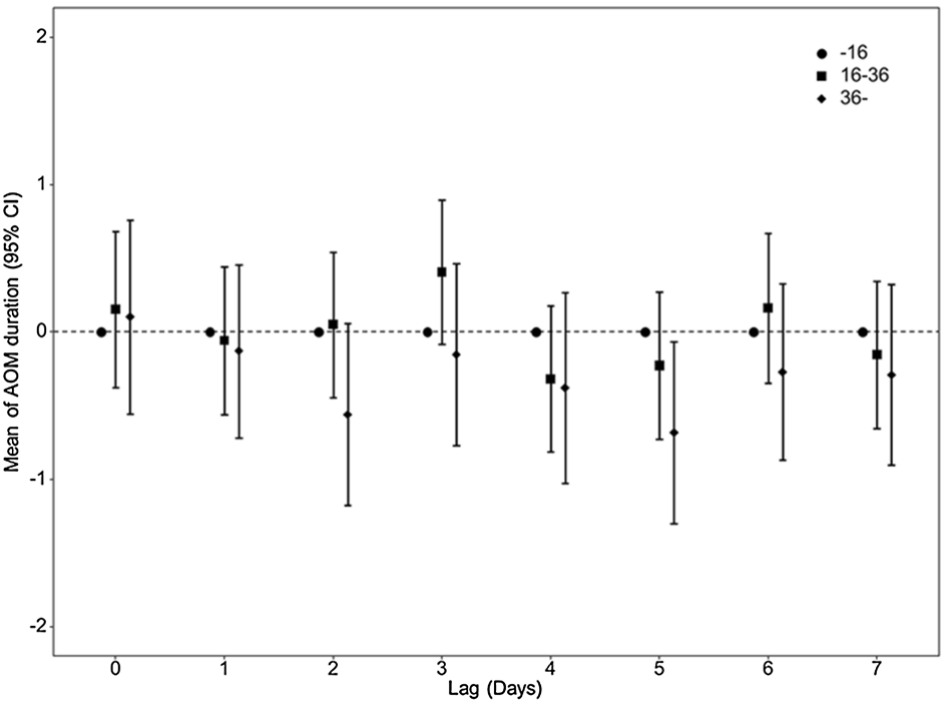
The effects of PM_2.5_ levels on the duration of acute otitis media (AOM) in each time lag. *The value is calculated in comparison with 0–16 μg/m^3^ of PM_2.5_.

After confounders or covariates were adjusted for, we analyzed the influence of the levels of PM_2.5_ on AOM duration at all time lags (Fig. 4). We also found no effects of PM levels on antibiotic use in AOM patients (S. Fig. 3).

## Discussion

In this study, we demonstrated that PM exposure was associated with the development of AOM. The association was clear in children aged < 2 years for both PM_2.5_ and PM_10_ on the day of exposure. However, regardless of the size of PM, it did not affect disease duration and antibiotics usage. For PM_2.5,_ there was no definite effect on AOM on the same day of exposure between urban and rural areas. This study used the national sample cohort database, which comprises data on about 1 million individuals sampled from the whole Korean population of about 51 million^20^. To our knowledge, this study includes the largest study population to investigate the association between OM and PM exposure.

AOM development after PM exposure has been demonstrated in several in vitro and in vivo experiments. An in vitro study has shown that PM stimulates the development of OM by promoting apoptosis, the expression of inflammatory cytokines (TNF-α and COX-2), and the expression of a mucin gene (Muc5AC)^27^. In an in vivo study, injection of PM into the middle ear of Sprague Dawley rats increased the thickness of the middle-ear mucosa and the infiltration of inflammatory cells^28^. A transcriptomic analysis of mice exposed to diesel exhaust particles demonstrated the upregulation of genes related to IL-2 expression and T-cell maturation and the downregulation of CD4, IFNA1, and ESR1^29^*.*

In this study, we demonstrated that children younger than 2 years were affected by PM exposure regardless of its diameter. Our previous study used national sample cohort database–enrolled children aged > 5 years because the data were not classified by whether the subjects were older or younger than 2 years of age^13^. Children are highly susceptible to the deleterious effects of environmental pollution because they are more physically active than adults and have higher respiratory rate and hence enhanced deposition of pollutants^30–32^. Moreover, because the function of the Eustachian tube is insufficient in children compared to that in adult, the incidence of OM is higher in young children than in adults^33,34^. The rapid development of the Eustachian tube and temporal bone occurs in the first 2 years of life^35^.

We found an association of OM development with the same day of PM exposure in children aged < 2 years. On the other hand, Kousha et al. reported that PM_2.5_ is associated with the number of emergency department visits in OM children and found the highest OR from a lag of 3 days^36^. Xiao et al. also reported increment in emergency room visits with OM diagnoses 3–4 days after exposure to higher PM in a case-crossover study in Georgia, USA^37^. Most of our data might consist of more mild-symptom cases than aforementioned studies. This is because our study includes visits to a primary care physician, not emergency department. It is easier to visit a primary care physician than to visit emergency department. In addition, owing to the national insurance system in Korea, the access to clinics/ hospitals is easy and medical cost is low. These factors may explain the difference in lag time between our and aforementioned studies.

In 2020, Oh et al.^38^ published a paper quite similar to this study. Both studies aimed to evaluate the association between PM levels and AOM occurrence in South Korea using the same data sources (NHIS and Korean National Institute of Environmental Research), and for a similar period. However, there are some differences: (1) they surveyed children under 3 years of age, whereas we surveyed children under 10 years of age, with three age subgroups; (2) they research included only for PM_2.5_ vs. both PM_2.5_ and PM_10_ in our study; (3) their survey used moving day (the average of day 0, 1, 2, 3, and 4 days ago) to show the pooled exposure–response, whereas we used not only pooled data, but also each day (day 0, 1, 2, 3, 4, 5, 6, and 7 days ago) to reveal delayed effects; (4) their research included only seven major cities vs. 16 administrative regions including rural and urban regions in our study.

We could not analyze the incidence according to sex and age groups for all regions because of limited data, but did so for the two largest regions (Gyeonggi and Seoul). Our study showed higher incidences of AOM in girls exposed to PM_2.5_ and boys exposed to PM_10_ for children under 2 years. Some studies have found a higher incidence and more recurrence of AOM in boys^39–41^. On the other hand, most studies have found no difference in incidence according to sex^42–45^. Generally, AOM is known to be more common in urban areas because of air pollution and high population density. We found no difference in the association of OM development with PM exposure between urban and rural areas. This may due to the small size of the country and its high urbanization level. The slope of the concentration–response curve of PM-induced mortality is steeper at low mean concentration than at high mean concentration^46^. Korea has higher mean PM concentration than other countries^13,37,47–49^, and this could be one reason. Finally, we could not directly compare which size of PM is more harmful to AOM because of the difference in data range for PM_2.5_ and PM_10_. Generally, PM_2.5_ is more harmful for health than PM_10_^46^. Smaller particles can reach deeper into the respiratory system and penetrate down to the alveoli by diffusion^7,50^.

As far as we know, this is the first study to evaluate the duration of AOM and antibiotic use after PM exposure. Unfortunately, we did not find any definite relation between PM levels and duration of AOM, probably because the incidence of AOM is too low to show the association. Whether to prescribe antibiotics or not (careful waiting) for AOM has been controversial for a long time. Recent guidelines and most ongoing studies recommend careful waiting in children with non-severe AOM^51–53^. This is why we found no relation between PM exposure and antibiotic use.

To increase the accuracy of the data in this study, we paid careful attention to the following concerns. The terminology concerning OM is quite confusing. In AOM, the acute, suppurative infectious course is characterized by the presence of infected middle ear fluid and inflammation of the middle ear mucosa^53^. In this study, we clearly distinguished between these findings and OM effusion, fluid in the middle ear that was not infected, and excluded the latter^54^. Moreover, we defined AOM according to the ICD-10, which was established in May 1990 by the Forty-43rd World Health Assembly. ICD-10 has been mentioned in more than 20,000 scientific articles and used by more than 150 countries around the world and has international reliability. Korean Standard Classification of Disease (KCD) version 6 is based on the ICD-10 and diagnoses in NHIS were coded according to the KCD-6^55^.

This research has some limitations. First, the vaccine status, especially for *Streptococcus pneumoniae*, is a decisive factor for the development of AOM^56,57^, but it was not investigated. Taking the vaccine status into account in our next study will certainly strengthen it. Second, we could not be confident whether the new guidelines, which recommend antibiotics only for certain cases of AOM, were followed in all cases because of the time discrepancy between the study period and the development of the guidelines^22^.

## Conclusions

Regardless of PM size and children’s age, PM levels increase the incidence of AOM. This effect is clearest in children under 2 years of age and on the day of PM exposure.

## Supplementary Information

Supplementary Information.
