# Supplementary material for: Particular matter influences the incidence of acute otitis media in children
Source: Sci Rep. 2021 Oct 5;11:19730. doi: 10.1038/s41598-021-99247-3 (PMC8492675; doi:10.1038/s41598-021-99247-3)
Supplement: Supplementary file 1 — Supplementary Information. [file 41598_2021_99247_MOESM1_ESM.pdf]

Particular Matter Influences the Incidence of Acute Otitis Media in Children

Mina Park[1], Jiyeon Han[2], Jiwon Park[2], Myoung-jin Jang[2], Moo Kyun Park[3,*]

[1]Department of Otorhinolaryngology-Head and Neck Surgery, Seoul Medical Center, Seoul, Korea.

[2]Medical Research Collaborating Center, Seoul National University Hospital, Seoul, Korea.

[3]Department of Otorhinolaryngology-Head and Neck Surgery, Seoul National University College of Medicine, Seoul, Korea.

Running Head: Association between Particular Matter and Otitis Media in Children

Correspondence to: *Moo Kyun Park, M.D., Ph.D.*

Department of Otorhinolaryngology-Head & Neck Surgery, Seoul National University College of Medicine, Seoul National University Hospital

101 Daehangno, Jongno-gu, Seoul, South Korea, 03080

Tel: 82-2-2072-2446; Fax: 82-2-745-2387

E-mail: aspetic@snu.ac.kr

		Daily AOM incidence (per 1,000 person)			Daily level of $PM_{2.5}$		Daily level of PM_{10}	
		n	Mean (SD)	Median (Q1, Q3)	Mean (SD)	Median (Q1, Q3)	Mean (SD)	Median (Q1, Q3)
Region	Gangwon	5601	2.5 (1.7)	2.4(1.3,3.6)	46.4 (26.7)	41.5(28.9,56.9)	26.4 (14.3)	23.8(15.3,35)
	Gyeonggi	50092	3.4 (1.7)	3.5(2.3,4.6)	55.4 (30.6)	50.1(36.1,66.9)	25.4 (13.6)	22.5(15.8,32.7)
	Gyeongman	13123	2.5 (1.5)	2.4(1.4,3.4)	46.3 (23.7)	41.9(31.1,55.0)	25.4 (10.9)	24.4(17.3,31.4)
	Gyeongbuk	9938	2.8 (1.6)	2.8(1.7,3.8)	46.8 (23.5)	41.9(31.4,55.7)	21.8 (12.1)	19.0(14.0,26.0)
	Gwangju	5929	2.7 (1.6)	2.5(1.4,3.6)	43.3 (27.1)	38.0(27.0,52.2)	26.1 (14.0)	23.5(15.7,32.5)
	Daegu	8831	2.3 (1.5)	2.1(1.2,3.1)	47.5 (26.2)	42.5(31.1,57.4)	25.3 (12.3)	22.7(16.1,32.4)
	Daejeon	6429	2.7 (1.7)	2.7(1.3,3.8)	43.0 (25.3)	38.5(26.4,53.1)	29.0 (16.4)	25.0(16.0,39.0)
	Busan	11254	1.9 (1.2)	1.8(1.0,2.7)	47.7 (24.4)	42.1(32.6,57.0)	25.5 (11.4)	23.8(16.9,32.4)
	Seoul	35911	2.9 (1.6)	3.0(2.0,4.0)	47.8 (30.3)	42.1(29.4,58.0)	23.1 (11.5)	21.2(15.9,28.4)
	Ulsan	4728	2.9 (1.9)	2.8(1.6,4.1)	47.9 (25.3)	42.2(31.1,59.2)	24.6 (12.5)	22.3(14.3,31.8)
	Incheon	11276	3.0 (1.7)	3.0(1.9,4.1)	52.4 (29.4)	46.5(34.1,62.6)	28.5 (14.3)	25.3(18.2,36.4)
	Jeonnam	7080	2.7 (1.5)	2.5(1.6,3.7)	40.3 (22.8)	35.5(26.7,47.5)	24.9 (12.1)	22.8(16.0,31.0)
	Jeonbuk	6814	3.2 (1.8)	3.2(1.9,4.3)	50.0 (26.0)	45.4(32.9,60.8)	34.3 (20.2)	31.2(18.5,43.7)
	Jeju	2416	4.0 (2.6)	3.7(2.2,5.7)	43.0 (30.1)	36.0(27.3,50.0)	23.2 (13.7)	20.5(13.5,29.5)
	Chungman	8793	2.7 (1.6)	2.6(1.5,3.7)	45.5 (26.1)	40.7(29.3,55.0)	27.0 (15.3)	23.0(18.0,34.0)
	Chungbuk	5967	2.9 (1.8)	2.8(1.4,4.1)	54.4 (30.0)	49.6(34.0,67.9)	27.1 (18.3)	22.5(13.5,38.4)
Age	<2	83248	4.1(2.5)	3.9(2.4,5.6)	-	-	-	-
	2-4	92282	4.7(2.5)	4.6(3.1,6.3)	-	-	-	-
	5-10	122698	1.4(0.8)	1.5(0.9,2.0)	-	-	-	-
Total		169080	2.9(1.5)	3.0(2.0,3.9)	49.4(25.0)	44.9(33.0,58.6)	25.3(11)	24.5(17.4,31.4)

S. Table 1. The daily incidence of AOM per 1,000 children and levels of $PM_{2.5}$ and PM_{10}

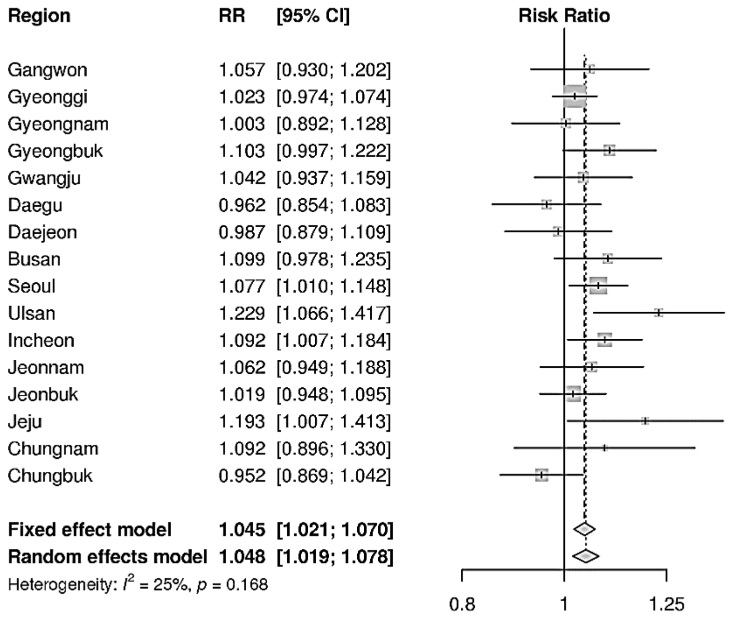

S. Figure 1. The diversity of relative risks of $PM_{2.5}$ in the 16 administrative regions.

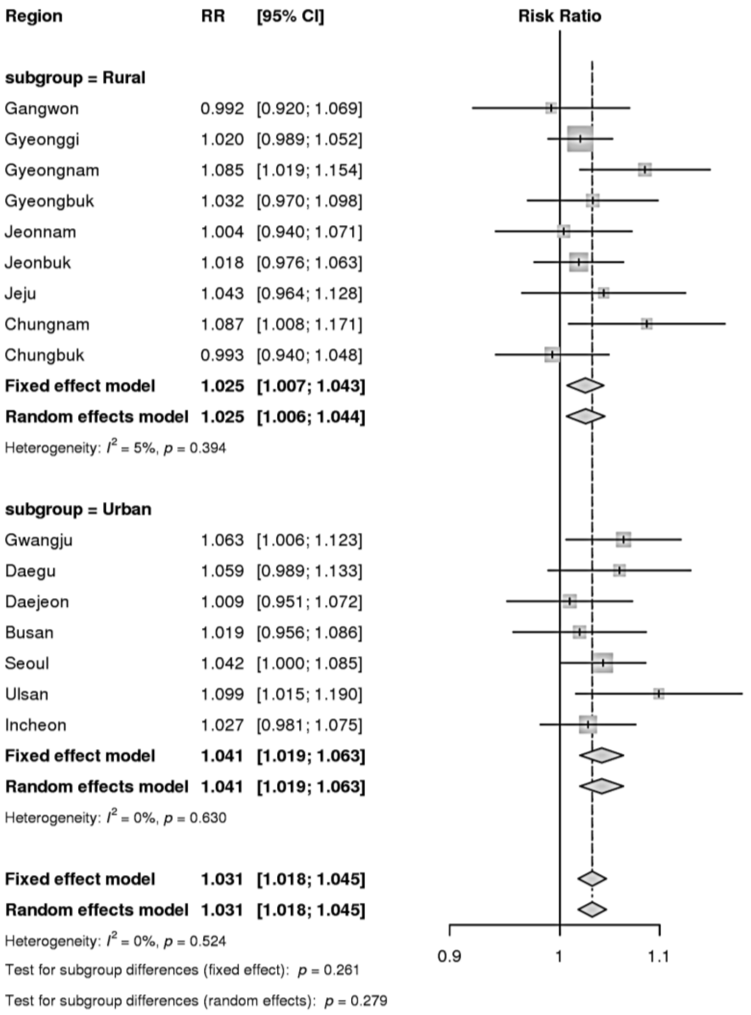

S. Figure 2. The influences of $PM_{2.5}$ to AOM on the day of exposure (lag 0) between rural and urban areas (RR = 1.025, 95% CI=1.007–1.043; RR = 1.041, 95% CI=1.019–1.063).

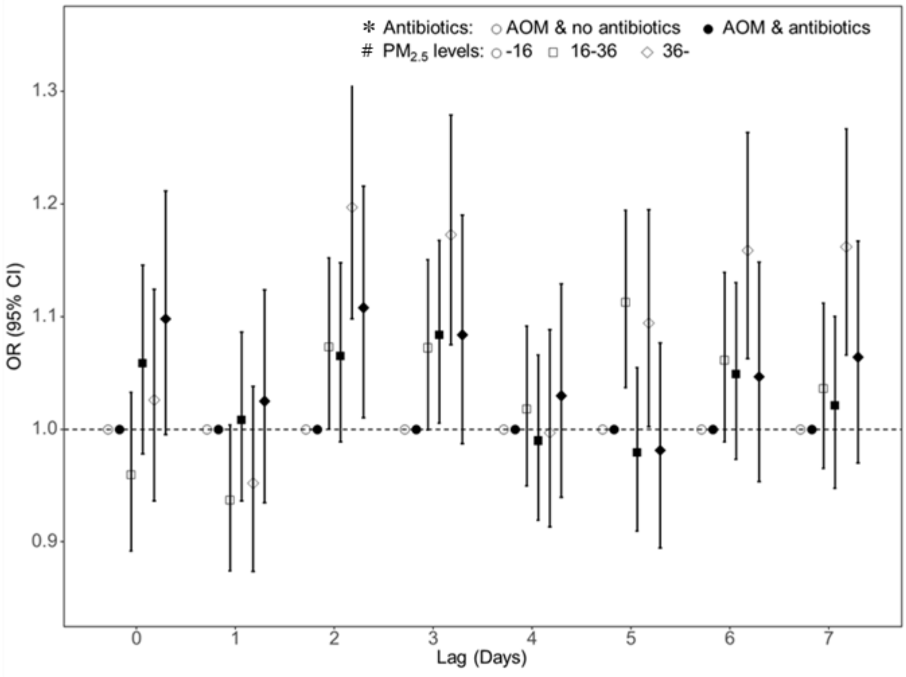

S. Figure 3. The effects of PM levels on antibiotic use in patients.

AOM: acute otitis media

* The value is calculated in comparison with the normal group.
[#] The value is calculated in comparison with 0–16 μg/m^3.
